# EEG Emotion Recognition Network Based on Attention and Spatiotemporal Convolution

**DOI:** 10.3390/s24113464

**Published:** 2024-05-27

**Authors:** Xiaoliang Zhu, Chen Liu, Liang Zhao, Shengming Wang

**Affiliations:** National Engineering Research Center of Educational Big Data, Central China Normal University, Wuhan 430079, China; zhuxl@ccnu.edu.cn (X.Z.); chenliu@mails.ccnu.edu.cn (C.L.); liang.zhao@ccnu.edu.cn (L.Z.)

**Keywords:** EEG, SEED, emotion recognition, graph neural network, 3D convolution

## Abstract

Human emotions are complex psychological and physiological responses to external stimuli. Correctly identifying and providing feedback on emotions is an important goal in human–computer interaction research. Compared to facial expressions, speech, or other physiological signals, using electroencephalogram (EEG) signals for the task of emotion recognition has advantages in terms of authenticity, objectivity, and high reliability; thus, it is attracting increasing attention from researchers. However, the current methods have significant room for improvement in terms of the combination of information exchange between different brain regions and time–frequency feature extraction. Therefore, this paper proposes an EEG emotion recognition network, namely, self-organized graph pesudo-3D convolution (SOGPCN), based on attention and spatiotemporal convolution. Unlike previous methods that directly construct graph structures for brain channels, the proposed SOGPCN method considers that the spatial relationships between electrodes in each frequency band differ. First, a self-organizing map is constructed for each channel in each frequency band to obtain the 10 most relevant channels to the current channel, and graph convolution is employed to capture the spatial relationships between all channels in the self-organizing map constructed for each channel in each frequency band. Then, pseudo-three-dimensional convolution combined with partial dot product attention is implemented to extract the temporal features of the EEG sequence. Finally, LSTM is employed to learn the contextual information between adjacent time-series data. Subject-dependent and subject-independent experiments are conducted on the SEED dataset to evaluate the performance of the proposed SOGPCN method, which achieves recognition accuracies of 95.26% and 94.22%, respectively, indicating that the proposed method outperforms several baseline methods.

## 1. Introduction

Human emotions are complex psychological and physiological responses, which are natural reactions to external stimuli that can cause various physiological changes in the body, e.g., accelerated heartbeat and sweating. Emotions play a crucial role in human cognition, especially in rational decision-making processes, perception, and interpersonal communication.

Currently, emotion recognition can primarily be divided into two categories. The first approach is the recognition of non-physiological signals, e.g., facial expressions, voice tone, and body posture. However, these signals can be controlled artificially through disguise and other means; thus, it is difficult to identify true emotional states accurately. The second approach employs physiological signals, e.g., EEG, electrooculogram, electrocardiogram, and electromyography signals, to recognize human emotions [1,2,3]. Physiological signals are difficult to disguise and can reflect human emotional states accurately; thus, such signals can be used to obtain more objective and realistic results. Among these physiological signals, EEG signals are spatially discrete nonstationary random signals generated by the central nervous system that can record changes in scalp potential directly. Compared with other physiological signals, EEG signals can more accurately and reliably reflect a person’s emotional state [4].

**Figure 1:**
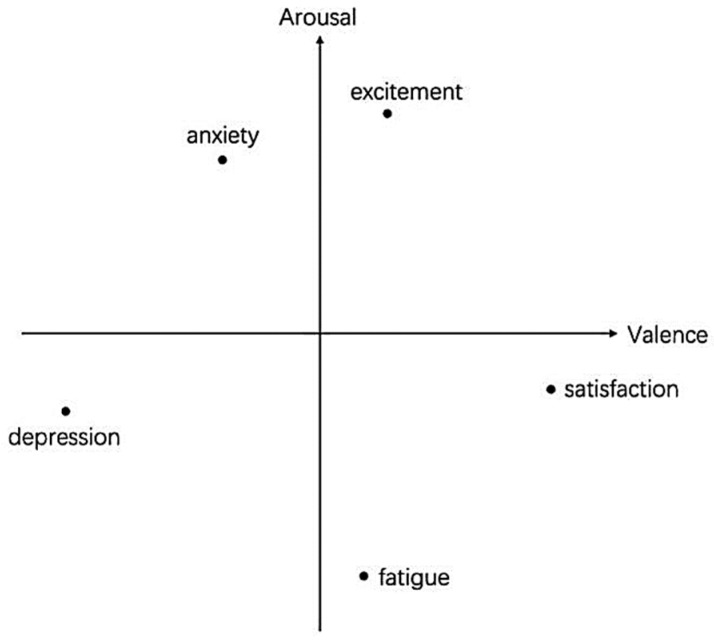
Two-dimensional spatial representation of emotions.

The first challenge in emotion recognition is determining how to categorize the emotions. There are two main types of emotion quantification models, i.e., discrete models and continuous models. Discrete models consider complex emotions as combinations of a limited number of basic emotions. For example, renowned psychologist Ekman proposed six basic emotion categories, i.e., anger, disgust, fear, happiness, sadness, and surprise, which have been widely accepted in the emotion recognition field [5]. However, as emotional research deepens, it is recognized that emotions are a continuous process. Thus, a dimensional model has been proposed, and this model divides the emotional space into two, three, or more dimensions based on cognitive evaluations to describe human emotions in a continuous form. Different emotional states are distributed in different positions in the dimensional space according to their attributes. The most widely used dimensional model is the valence arousal (VA) two-dimensional (2D) spatial model [6]. Here, the valence dimension reflects the positive or negative degree of emotions, transitioning from negative to positive from low to high, and the arousal dimension reflects the intensity of the emotions. In addition, there is a mapping relationship between the discrete model and the dimensional model. The distribution of five emotions, i.e., excitement, anxiety, depression, fatigue, and satisfaction, in the VA model is shown in Figure 1.

The input forms of EEG emotion recognition models can generally be divided into four categories, i.e., manually designed traditional features, raw or preprocessed signals, encoded image features, and other features.

Currently, most studies employ manually designed EEG features as inputs to the model, and these features can be categorized into three groups, i.e., time domain, frequency domain, and time–frequency domain features.

Typically, EEG signals are collected in the time domain, which makes time-domain features the most intuitive and easily obtainable. The time-domain characteristics of EEG signals have been studied extensively in EEG research. Representative examples of commonly used time-domain features include event-related potentials, signal statistics, Hjorth parameter features, and fractal dimension features. For example, Kashihara obtained event-related potentials by stimulating subjects and used statistical features, e.g., the signal mean and standard deviation, as EEG features [7]. Tripathi et al. extracted features, e.g., skewness and kurtosis, from EEG signals in the DEAP dataset and conducted sentiment recognition research using deep neural networks (DNNs) and CNNs in both the valence and arousal dimensions, achieving good recognition results [8].

Frequency domain features involve converting the original EEG signal from the time domain to the frequency domain and extracting relevant characteristics. Frequency domain analysis typically divides the EEG signals into the delta frequency band (1–3 Hz), theta frequency band (4–7 Hz), alpha frequency band (8–13 Hz), beta frequency band (14–30 Hz), and gamma frequency band (31–50 Hz) for feature extraction. Common frequency domain features include power spectral density and differential entropy (DE). For example, Al Nafjan combined power spectral density features extracted from EEG signals and employed DNNs to classify emotions [9]. In addition, Li et al. employed short-time Fourier transform (STFT) for time–frequency conversion, calculated power spectral density features and facial expression features in the theta, alpha, beta, and gamma frequency bands, fused them, and then used a long short-term memory network for emotion recognition, achieving good recognition results [10].

Time–frequency characteristics involve segmenting signals with sliding windows and performing time–frequency domain signal transformations on each sub-signal segment. This method combines the advantages of time-domain and frequency-domain analyses to improve the processing ability of unstable signals. Commonly used time–frequency analysis methods include STFT, wavelet transform (WT), and Hilbert Huang transform (HHT). Chen et al. improved classification and recognition accuracy by employing a time–frequency domain sentiment feature analysis method based on the reconstruction of EEG signal sources [2].

Early recognition algorithms were primarily based on traditional machine learning methods, e.g., support vector machines (SVM), k-nearest neighbors, random forests, and naive Bayes. However, these methods frequently involve using manually extracted features as inputs, which is a time-consuming and labor-intensive process that can result in information loss. In addition, EEG signals are susceptible to noise interference during the signal acquisition process. EEG signals also have a low signal-to-noise ratio and exhibit time-based asymmetry and instability. These unique features present challenges for traditional machine learning methods that are heavily dependent on manual feature extraction processes and prior knowledge.

Given the remarkable success of deep learning methods in various recognition tasks, researchers have begun utilizing various deep learning frameworks for the task of EEG-based emotion recognition. Compared with traditional machine learning methods, deep learning techniques offer stronger capabilities in terms of describing and fitting problems. Current research efforts are focused on investigating the impact of different brain functional regions, frequency band combinations, and time-domain features on emotion classification performance. In addition, neurological research has revealed complex functional connections among different brain regions during emotion generation, which has prompted researchers to investigate the relationships between these regions. For example, Ding et al. introduced the Local Global Graph Representation Learning (LGGNet) technique for brain–computer interfaces which employs both local and global graph filtering layers to gain insight into the brain activity in and between different functional regions [11] Experimental results indicated emotional asymmetry and abundant emotional information in the frontal lobe. Subsequently, Ding et al. proposed a multiscale convolutional neural network (CNN) called Tsception, which effectively captures the temporal dynamics and spatial asymmetry of EEG signals by learning discriminative representations in both the time and channel dimensions [12]. The experimental findings suggested that the right hemisphere of the brain plays a unique role in emotional processes. In addition, Li et al. proposed a self-organizing graph neural network (SOGNN) for cross-subject EEG emotion recognition [13]. This SOGNN comprises three convolutional pooling blocks, three self-organizing layers, three graph convolutional layers, a fully connected layer, and an output layer. The experimental results demonstrate the effectiveness of models with sparse neighbor matrices. Zhong et al. proposed a regularized graph neural network (RGNN) for emotion recognition based on electroencephalography [14]. This model considers the biological topology between different brain regions to capture local and global relationships among different EEG signal channels, and the experimental results demonstrate the importance of global connectivity when modeling differential asymmetry in electroencephalography. Wang et al. proposed a feature fusion method that can effectively reflect emotional states [15]. This method is characterized by multichannel weighted multiscale permutation entropy, which considers the time–frequency and spatial information of EEG signals and eliminates the inherent volume effect of EEG. Many previous studies have investigated the relationship between EEG frequency bands and emotion types. For example, Zhu et al. proposed an EEG signal emotion classification network based on multichannel frequency band feature attention fusion, which focuses on the relationship between frequency bands, channels, and time-series features [16]. The experimental results indicated that the proposed attention fusion unit generally has better performance for more frequency band combinations. Li et al. proposed a spatial frequency convolutional self-attention network (SFCSAN) that combines spatial and frequency domain feature learning from EEG signals [17]. This model employs intraband self-attention to learn the frequency information for each frequency band, and interband mapping further maps the smallest attention representation to learn supplementary frequency information. As a temporal signal, some researchers have found that the spatial information of multiple electrodes on a time slice and the contextual dependence of EEG signals are crucial for effective emotion recognition. In addition, Tao et al. proposed an attention-based convolutional recurrent neural network (ACRNN) that uses a channel attention mechanism to allocate weights adaptively to different channels and uses a CNN to extract the spatial information of the encoded EEG signal [18]. The results demonstrate that the neural network combines channel attention modules with extended self-attention, which can utilize more discriminative information for EEG-based emotion recognition. Wang et al. proposed a spatiotemporal recurrent neural network (STRNN) that integrates spatiotemporal information feature learning of signal sources into a unified spatiotemporal dependency model [15]. This STRNN can learn the spatial correlation of multiple electrode or image contexts and long-term memory information in time series.

Based on the above research, it has been determined that both spatial correlation and temporal contextual information play a crucial role in analyzing emotions using EEG signals. In line with the relevant research, we propose an EEG emotion recognition network that incorporates attention and spatiotemporal convolution. The spatial relationships between electrodes vary across frequency bands; thus, the proposed method first constructs a self-organizing graph for each channel in each frequency band. This graph identifies the 10 most relevant channels to the current channel and employs graph convolution to capture the spatial relationships between channels within each frequency band. Then, the proposed method employs pseudo-3D convolution and partial dot product attention to extract temporal features. Finally, LSTM is employed to learn the contextual information between adjacent time series. To evaluate the performance of the proposed SOGPCN method, we compare it with several state-of-the-art deep and nondeep methods in the BCI field, including SVM, TCA, SA, DGCNN, DAN, BiDANN-S, BiHDM, RGNN, and other methods on the SEED dataset.

The primary contributions of this study are summarized as follows.

1. To obtain the spatial features of the brain and make the spatial relationships of each frequency band relatively independent, we construct a self-organizing map for each channel within each frequency band. This map retains the 10 most relevant channels for each channel, and then we extract the spatial relationships between all electrodes in each frequency band through graph convolution.

2. To reduce model complexity while maintaining sufficient performance, we employ pseudo-3D convolution to extract temporal features from the extracted spatial features of the EEG signals. Here, we select 12 channels suitable for emotion classification.

3. To make the model focus more on the parts of the sequence that are relevant to emotion recognition, we employ a combination of dot product attention and pseudo-3D convolution to assign weights to the relevant information of the EEG sequence.

The remainder of this paper is organized as follows. Section 2 provides an overview of SOPCN-related technologies and Section 3 describes the architecture and implementation of the proposed SOPCN model. Section 4 discusses experiments and analyzes the results and performance of the proposed SOPCN model. Finally, the paper is concluded in Section 5, including a discussion on prospects for future research.

## 2. Related Works

### 2.1. Input Features of EEG Data

Previous research suggests that DE features are particularly discriminative for the task of emotion recognition. Therefore, for the proposed model, we employ DE features as input features. A DE feature is a frequency domain feature calculated through 512-point STFT, with a 1 s nonoverlapping Hanning window averaged across five frequency bands. Thus, the output DE features can be represented as a 5 × T matrix, where T represents the time window dependent on the stimulated film clip. The time window T of the SEED dataset is 185–265(in s). For the normalized reference SOGNN, features with short time windows are filled with zeros to the length of 265 in the SEED dataset. The input EEG signal feature size of the model is calculated as electrode × frequency band × time frame, and the input form of the data in the SEED dataset is 12 × 5 × 265.

### 2.2. CNNs for EEG Data

Recently, CNNs have demonstrated good performance in EEG research. Tao et al. proposed an ACRNN that utilizes a channel attention mechanism to assign weights adaptively to different channels and uses a CNN to extract the spatial information from the encoded EEG signals [18]. However, a limitation of the spatial feature extraction module is that the use of attention mechanisms to assign weights to different channels only considers the importance of different channels in the brain’s emotional generation process. In other words, the connectivity between brain channels is not considered. Xiao et al. proposed an attention-based four-dimensional (4D) neural network EEG emotion recognition method [19]. The overall structure of this method comprises a 4D spatial spectral temporal representation, an attention-based CNN, an attention-based bidirectional LSTM network, and a classifier. To utilize the spatial relationships of electrodes, the authors organized all channels into a 2D map, which limits the dimensionality of the data and hinders full utilization of the complex topological structure of brain signals. Graph neural networks (GNN) attempt to construct neural networks using graph theory to process data in the graph domain. The graph CNN (GCNN) is an extension of traditional CNN methods that combines a CNN with spectral theory. Compared to conventional CNN methods, the GCNN has advantages in terms of extracting discriminative features of the signals in the discrete spatial domain. More importantly, the GCNN method provides an effective mechanism to describe the intrinsic relationships between different nodes in the graph, which may provide a potential method to explore the relationships between multiple EEG channels in the process of EEG emotion recognition.

Two-dimensional CNNs are commonly used for image recognition tasks because they can extract spatial features effectively. In comparison, 3D CNNs can capture both spatial and temporal features simultaneously. In the biological signal analysis field, some 3D CNN models have been applied to epilepsy seizure prediction and motion image analysis tasks. However, there is relatively little research on applying 3D CNNs to the task of EEG-based emotion recognition. We use pseudo-3D convolution to extract the temporal and spatial features of EEG signals. Compared with ordinary 3D convolution, pseudo-3D convolution saves a lot of computational resources.

The GNN is a deep learning model used to process graph-structured data, e.g., molecular networks, social networks, and knowledge graphs [20]. EEG signals can be considered a typical type of graph-structured data. Recently, many studies have demonstrated the effectiveness of GNNs in processing brain signals. For example, Song et al. proposed a dynamic graph CNN (DGCNN) for emotion recognition from EEG signals [21]. This model employs a graph to capture the features of multichannel EEG signals. The graph structure is determined by a dynamic adjacency matrix that reflects the intrinsic relationships between the different EEG electrodes. The proposed method effectively characterizes the intrinsic relationships between EEG channels and achieves an accuracy of 79.95% on the SEED dataset. Zhong et al. proposed a regularized GNN (RGNN) for emotion recognition based on electroencephalography [14] This model considers the biological topology between different brain regions to capture the local and global relationships between different EEG signal channels. This previous model achieved an accuracy of 85.30% on the SEED dataset. This paper uses a self-organizing graph structure to explore the connectivity structure between different pathways in the brain.

### 2.3. Channel Selection

Most existing EEG data are collected using as many electrodes as possible to ensure comprehensive signal collection. These signals may include both emotion-related information and some interference signals or noise. According to Ding et al., the frontal lobe of the brain is rich in emotional information, and the temporal lobe is associated with emotional processes [11]. Zhong et al. discovered that the prefrontal, parietal, and occipital regions might contain the most abundant emotional recognition information [14]. Observing experimental results, Li et al. found that the left and right temporal lobes contribute more to emotion recognition [22]. Zheng et al. designed different electrode arrangements based on the peak and mismatch characteristics of the weight distribution in emotional processing [23]. These include four channels (FT7, FT8, T7, and T8), six channels (FT7, FT8, T7, T8, TP7, and TP8), nine channels (FP1, FPZ, FP2, FT7, FT8, T7, T8, TP7, and TP8), and 12 channels (FT7, T7, TP7, P7, C5, CP5, FT8, T8, TP8, P8, C6, and CP6). By employing DE features, an SVM method achieved good classification results on 12 channels, with an average accuracy of 86.65%. In the proposed method, we refer to the selection method of Zheng [23] for 12 channels and conduct comparative validation experiments on the SEED dataset using both 12 and 62 channels.

## 3. Proposed Methodology

**Figure 2:**
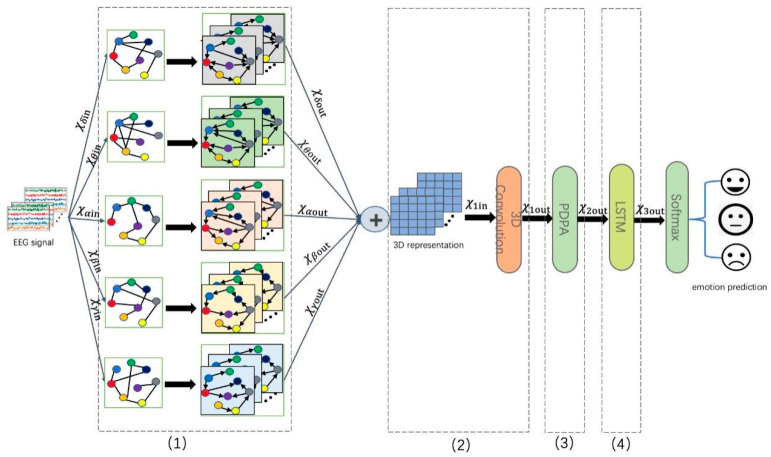
Overall architecture of the proposed SOGPCN method.

The architecture of the proposed model, namely SOGPCN, is shown in Figure 2. SOGPCN primarily comprises a spatial feature extraction module (Module 1) and a temporal feature extraction module (Modules 2 and 3). The input of the spatial feature extraction module utilizes DE features from the SEED dataset, where the input form of the data is 12 × 5 × 265. Initially, the data from the five frequency bands in the original data are separated, and a self-organizing graph structure is constructed for each frequency band. Here, graph convolution is employed to extract the spatial relationship between the electrodes in each frequency band, which ensures that the spatial features of the different frequency bands are not mixed, thereby preserving the respective graph structures. Then, the data from the five frequency bands are combined and reorganized into a batch size × channels × 5 × 64 × 1 format prior to being input to the temporal feature extraction module. In the temporal feature extraction module, the input data undergo one 3D convolution and two pseudo-3D convolutions. In the 3D convolution process, a 3 × 3 × 5 convolution kernel is employed with a convolution stride of 1 × 1 × 3 using zero padding and ReLU activation functions. Note that the operations of the two pseudo-3D convolutions are identical, utilizing a 3 × 3 × 1 convolution kernel and a 1 × 1 × 3 convolution kernel, respectively, with both employing zero padding and ReLU activation functions. After each pseudo-3D convolution, partial dot product attention is utilized, and average pooling with a size of 2 × 2 × 2 is performed. Following the extraction of the temporal features, the data are transformed into a one-dimensional (1D) tensor, and an LSTM network is used to learn contextual connections. Finally, the softmax function is employed for classification.

As shown in Figure 2, SOGPCN primarily comprises four modules: (1) a self-organizing graph convolution module is employed to extract the spatial features from each frequency band; (2) 3D-CNN layers are employed to extract temporal features from the multichannel EEG signals; (3) for each 3D-CNN layer, several dot product attention layers are implemented to help the model focus on valuable information; and (4) LSTM layers are implemented to learn the contextual information between adjacent time-series data. The input and output forms of each part of the data are shown in Table 1.

### 3.1. Dataset

The proposed SOGPCN method was evaluated on the publicly available SEED dataset, which was used for sentiment analysis using physiological signals. The SEED dataset [23] contains EEG data from 15 subjects (seven males and eight females) obtained using the ESI NeuroScan System at a sampling rate of 1 kHz. The EEG data were acquired from 62 channels. These data were collected while the participants were watching movies that evoked negative, neutral, and positive emotions. Each movie segment lasted approximately four minutes. The data collection process was divided into three sessions, with each session consisting of 15 segments of EEG data. Thus, a total of 45 segments of EEG data were collected for each participant. The data were downsampled to 200 Hz. A bandpass frequency filter from 0–75 Hz was applied. In the “Extracted_Features” folder, there are data on the differential entropy (DE) features of the extracted EEG signals, which we use for mood classification.

### 3.2. Self-Organizing GNN

In fact, EEG signals can be considered a typical type of structured data and are defined on a graph. Graph representation techniques and graph neural networks have been found to be very effective in processing EEG signals. The representation of EEG signals in the graph structure is expressed as follows.

(1) $$\begin{array}{l} G=(V,E,A)\\ V=\left\{v_{i}|i=1,\ldots ,N\right\}\\ E=\left\{e_{ij}|v_{i},v_{j}\in V\right\}\\ A=\left\{a_{ij}\right\} \end{array}$$

Here, V represents the N nodes in graph G, E represents the set of edges between nodes in V, $A\in R^{N\times N}$ represents the adjacency matrix, and $a_{ij}$ represents the connection weights of nodes $v_{i}$ and $v_{j}$, which indicates the relationship between the electrodes.

Li et al. proposed the SFCSAN, which considers that the spatial relationships between electrodes in each frequency differ [17]. The SFCSAN employs intraband self-attention to learn the frequency information of each frequency band, and interband mapping is employed to further map the smallest attention representation learning to learn additional frequency information. This network can model the intrinsic dependency relationships between EEG signal features in different frequency bands.

Thus, we utilize a self-organizing graph construction module to model the EEG sentiment features for each frequency band. Due to the diverse spatial and functional connectivity relationships between EEG signal channels, the closer the spatial relationship, the more it becomes less likely that there is a functional relationship. In this paper, we refer to the self-organizing map used by SOGNN [13], where the adjacency weights of the self-organizing map are defined as follows, by the function $f\left(v_{i},v_{j}\right)$.

(2) $$a_{ij}=f\left(v_{i},v_{j}\right)=\frac{\exp\;\left(\theta \left(v_{i}W\right)\theta {\left(v_{j}W\right)}^{T}\right)}{\sum_{i=1}^{N}\;\exp\;\left(\theta \left(v_{i}W\right)\theta {\left(v_{j}W\right)}^{T}\right)}$$

Here, $v\in R^{1\times F}$ is the eigenvector of a node in $V\in R^{N\times F}$, and $W\in R^{F\times L}$ and θ are the weights of the linear layer and the tanh activation function, respectively. The exponential function is a part of the softmax normalization activation function, which obtains a positive bounded adjacent weight.

The self-organizing adjacency matrix is calculated as follows.

(3) $$\begin{array}{c} G=\mathrm{Tanh}\;(VW)\\ A=\mathrm{Softmax}\;\left(GG^{T}\right) \end{array}$$

Here, $V\in R^{N\times F}$ is the input feature matrix of the node and $W\in R^{F\times L}$ is the weight matrix. The tanh activation function is employed to obtain the output $G\in R^{N\times L}$ of the linear layer. Then, the softmax function is applied to obtain a positive bounded adjacent matrix A. To reduce computational cost, sparse graphs are constructed using the top-k technique. Here, only the k largest weights of the adjacent matrix are preserved and small connection weights are set to zero. The top-k operation is performed as follows.

(4) $$\left\{\begin{array}{l} \;\mathrm{for}\;i=1,2,\cdots ,N\\ \mathrm{index}=argtopk\;(A[i,:])\\ A[i,\bar{index}]=0 \end{array}\right.$$

Here, the argtopk (·) function calculates the indices of the top-k maximum values in each vector A[i,:] of the adjacency matrix A. In this case, the $\bar{index}$ represents indices that do not belong to the top-k maximum values. Only the maximum k values are retained in each row vector of the adjacency matrix A and the remaining values are set to 0. In this paper, k is set to 10.

Based on the above analysis, the first step involves employing a self-organizing graph module to construct a graph structure for each frequency band based on the input EEG features. Then, a graph convolution layer is applied to process the constructed graph to extract the local and global connectivity features for emotion recognition. The dependency relationship between electrodes in different frequency bands can be modeled using self-organizing graphs and graph convolution modules. After extracting the spatial relationship of each frequency band, the vectors of the different frequency bands are connected and reorganized to obtain the output $\chi \in R^{N\times M\times T\times 1}$, where N is the number of electrodes, M is the number of frequency bands, and T is the time step calculation.

### 3.3. Pseudo-3D Convolution

**Figure 3:**
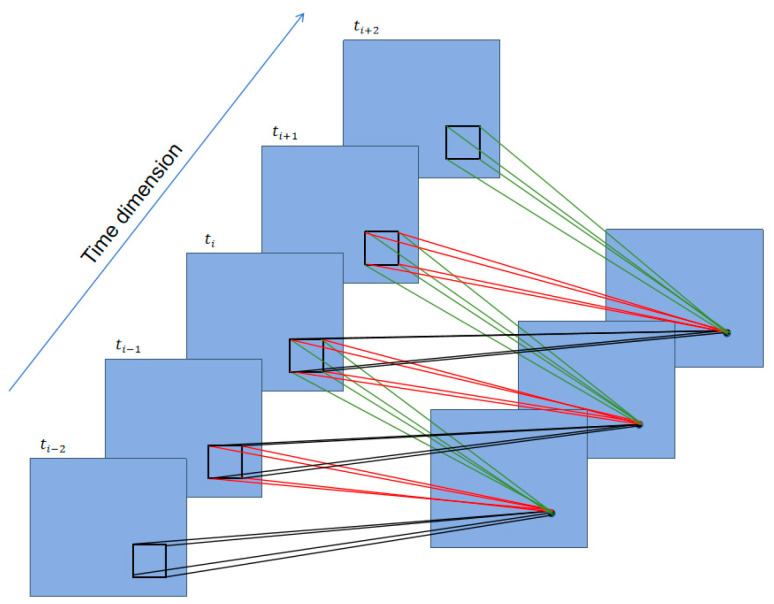
Example of 3D convolution on extracted 3D features.

3D convolution is achieved by applying a 3D convolution kernel to a 3D structure formed by stacking multiple continuous 2D feature maps over time, as shown in Figure 3. The 2D feature maps at time points $t_{t-2}$, $t_{t-1}$, $t_{t}$, $t_{t+1}$, and $t_{t+2}$ are stacked together using 3D convolution for feature extraction. Thus, compared to 1D temporal convolution and 2D spatial convolution, 3D spatiotemporal convolution has advantages in terms of characterizing brain connections and their activities. The values of the i-th and j-th layer feature maps at $\left(x,y,z\right)$ are calculated as follows [24].

(5) $$v_{ij}^{xyz}=tanh\;\left(b_{ij}+\sum_{m}\;\sum_{p=0}^{P_{i}-1}\;\sum_{q=0}^{Q_{i}-1}\;\sum_{r=0}^{R_{i}-1}\;w_{ijm}^{pqr}v_{(i-1)m}^{\left(x+p)(y+q)(z+r\right)}\right)$$

Here, $P_{i}$, $Q_{i}$, and $R_{i}$ are the sizes of the 3D convolution kernels in three dimensions, and $w_{ijm}^{pqr}$ is the value of the (p, q, r)-th kernel connected to the m-th feature map in the previous layer.

Although 3D convolution has considerable advantages in extracting temporal and spatial features, it incurs high computational costs. To address this issue, the proposed model adopts pseudo-3D convolution [25] to reduce computational complexity. The kernel size of standard 3D convolution is (P, Q, R), where P and Q denote the kernel sizes of the 2D spatial convolution, and R is the kernel size along the time dimension. In pseudo-3D convolution, the kernels (P, Q, R) are decoupled into P × Q × 1 and 1 × 1 × R, where P × Q × 1 represents a convolutional filter that is equivalent to 2D convolution in the spatial domain and 1 × 1 × R represents a convolutional filter that is equivalent to 1D convolution in the time domain. Thus, the output of the pseudo-3D module in the l-th layer can be defined as follows.

(6) $$\chi^{l}=\Phi^{1\times 1\times R}\left(\Phi^{P\times Q\times 1}\left(\chi^{l-1}\right)\right)$$

Here, $\chi^{l-1}$ is the output of layer l−1, and $\Phi^{P\times Q\times 1}$ and $\Phi^{1\times 1\times R}$ are 2D convolution kernels with a P × Q kernel in the spatial domain and 1D convolution kernels with an R kernel in the time domain, respectively.

### 3.4. Partial Dot Product Attention

An attention mechanism is a commonly used technique in deep learning that can make models more accurate and effective when processing sequence data. In traditional neural networks, the output of each neuron only depends on the output of all neurons in the previous layer. However, in attention mechanisms, the output of each neuron is dependent on the output of all neurons in the previous layer and can be weighted according to different parts of the input data. This means that different weights can be assigned to different parts, which allows the model to focus on key information in the input sequence. As a result, the accuracy and efficiency of the model can be improved. The proposed model utilizes the partial dot product attention mechanism from 3DsleepNet to quantify the importance of the input features. As a result, the most relevant information is assigned higher weights, and less relevant information is assigned lower weights. For a given input $\chi \in R^{N\times M\times T}$, the output of the partial dot product attention mechanism is defined as follows [26].

(7) $$Att=\chi ⊗\sigma \left(\left(\chi \cdot M_{1}\right)\cdot M_{2}+b\right)$$

Here, $M_{1}\in R^{T\times M}$, $M_{2}\in R^{M\times T}$, and $b\in R^{N\times M\times T}$ are learnable parameters, where ⋅ is the dot product and ⊗ is the dot product σ for the softmax function.

### 3.5. LSTM

An LSTM network is an RNN variant used to process sequence data. LSTM can learn long-term dependency relationships and overcome the gradient vanishing problem in traditional RNNs. As a type of sequence data, most studies have achieved superior performance using LSTM to process EEG signal data. The core concept of an LSTM network is to control the flow of information by introducing a gate structure. These gates include input gates, forget gates, and output gates, which determine the entry of new information, the forgetting of old information, and the output of information, respectively [27]. Through these gates, an LSTM network can determine which information needs to be remembered, which information needs to be forgotten, and how to update the state based on the current information. After performing partial dot product attention, the input χ is flattened as a 1D tensor. For a given input $\chi \in \left(x_{1},x_{2},x_{3},\ldots ,x_{n}\right)$, the output of the LSTM layer can be calculated as follows.

(8) $$\begin{array}{l} i_{t}=\sigma \left(W_{qi}q_{t}+W_{hi}h_{t-1}+W_{ci}C_{t-1}+b_{i}\right)\\ f_{t}=\sigma \left(W_{qf}q_{t}+W_{hf}h_{t-1}+W_{cf}C_{t-1}+b_{f}\right)\\ c_{t}=f_{t}C_{t-1}+i_{t}\tanh\;\left(W_{qc}q_{t}+W_{hc}h_{t-1}+b_{c}\right)\\ o_{t}=\sigma \left(W_{qo}q_{t}+W_{ho}h_{t-1}+W_{co}C_{t}+b_{o}\right)\\ h_{t}=o_{t}\tanh\;\left(c_{t}\right)\\ y_{t}=W_{ho}h_{t}+b_{o} \end{array}$$

Here, σ is the sigmoid function, i, f, o, and c are input gates, forget gates, output gates, and cell activation vectors, respectively, W is the weight matrix, and b is the bias vector.

## 4. Result

A series of experiments was conducted to evaluate the performance of the proposed model.

### 4.1. Experimental Setup

**Figure 4:**
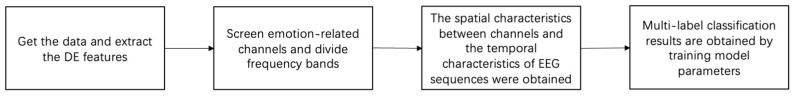
Experiment flowchart.

The proposed SOGPCN model was trained in Python using an NVIDIA GeForce GTX 1080 Ti GPU. The model’s loss was minimized using the Adam optimizer with a learning rate of 0.001 and a batch size of 15. The model was trained for 200 epochs using an early stop mechanism. During the training process, a dropout operation with a dropout rate of 0.1 was applied, thereby randomly blocking the output units of the inner layer. The experiment was mainly carried out in the following four steps, as shown in Figure 4: First, the SEED data set was obtained and DE features were obtained; second, emotion-related channels and frequency band division were screened; then, the spatial and temporal features between EEG channels were learned using the model; finally, multi-label classification results were obtained according to the trained model.

In this study, two experimental strategies, i.e., subject independence and subject dependence, were employed to validate the performance of the proposed model. The cross-validation strategy of keeping one subject independent was employed to evaluate the emotion recognition performance of each subject. In each experiment, data from 14 participants in the SEED dataset were used as the training dataset, and the data for the remaining participant were used as the validation dataset. We analyzed the average recognition accuracy of different subjects and frequency bands, and we compared and analyzed the impact of different frequency bands and channel combinations on the time complexity and recognition accuracy. The subjects relied on an experimental strategy, shuffling all the data and dividing it evenly into 15 parts, using 14 parts as the training dataset and the remaining part as the validation dataset. We also compared the performance of the model with other models to evaluate its effectiveness. A confusion matrix was provided to analyze data on different emotions. Finally, ablation experiments were conducted to demonstrate the effectiveness of different modules in the model.

### 4.2. Analysis of Experimental Results

#### 4.2.1. Independent Classification of Subjects

Table 2 shows the independent classification accuracy of the proposed model on the SEED dataset as well as its performance on both single and combined frequency bands. Here, we set the input features as a single frequency band and a combination frequency band. Here, many studies use (θ, α, β, and γ), and we conducted experiments on these four frequency bands [28] and provide corresponding experimental results. As shown in Table 2, the proposed model performs well on the δ, θ, α, β, and γ frequency bands. The accuracy obtained on these five frequency bands reached 91.56%, 91.11%, 82.81%, 89.78%, and 90.52%, respectively, thereby outperforming the baseline model [13,14,21,29,30,31,32] for all frequency bands. The combination of frequency bands (θ, α, β, γ) also demonstrated the best performance. For all emotion recognition methods, the average recognition accuracy was the highest when all five frequency bands were used simultaneously. In addition, on the SEED dataset, the proposed model achieved the lowest standard deviation in accuracy compared to all baseline models, demonstrating the robustness of the proposed model to cross-subject variations.

**Figure 5:**
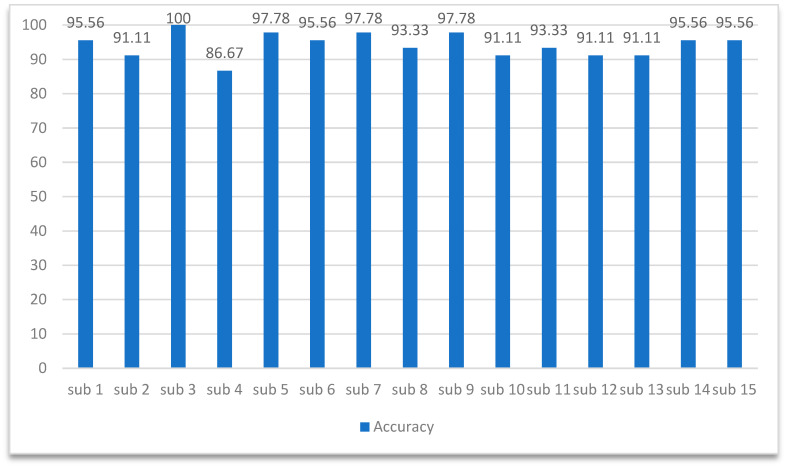
Analysis of each subject.

After obtaining the experimental results, we investigated the classification accuracy for each individual. The experimental results are shown in Figure 5. Except for the fourth participant, the accuracy of all other participants was greater than 90%, which proves that the proposed model has a certain degree of stability across different experimental subjects and has promising potential for practical applications.

#### 4.2.2. Subject Dependency Classification

Table 3 shows the classification accuracy of the proposed model for all subjects on the SEED dataset, as well as its performance on both single and combined frequency bands. Note that our models are located separately in the δ, θ, α, β, and γ frequency bands. As can be seen, the performance obtained on all five frequency bands is better than the baseline model [14,21,23,32,34,35]. In most cases, the recognition classification accuracy of the β and γ frequency bands was better than the other three frequency bands, which is consistent with the results reported by previous studies [14,16,21]. In addition, the proposed model outperformed all baseline models on the SEED dataset when combining the features of the θ, α, β, and γ frequency bands. On the α, β, and γ frequency bands, the standard deviation of the proposed model was much lower than that of the baseline model. By comparing the results shown in Table 2 and Table 3, it is easy to observe that the proposed model achieved nearly the same accuracy for both the subject-independent and subject-dependent experimental strategies. One possible reason for the narrowing of the performance gap is that the structure of the model is relatively simple, with a small number of parameters, which will not cause model overfitting even in the case of a small amount of data, and this results in a significant difference between the subject-dependent and subject-independent experimental results.

**Figure 6:**
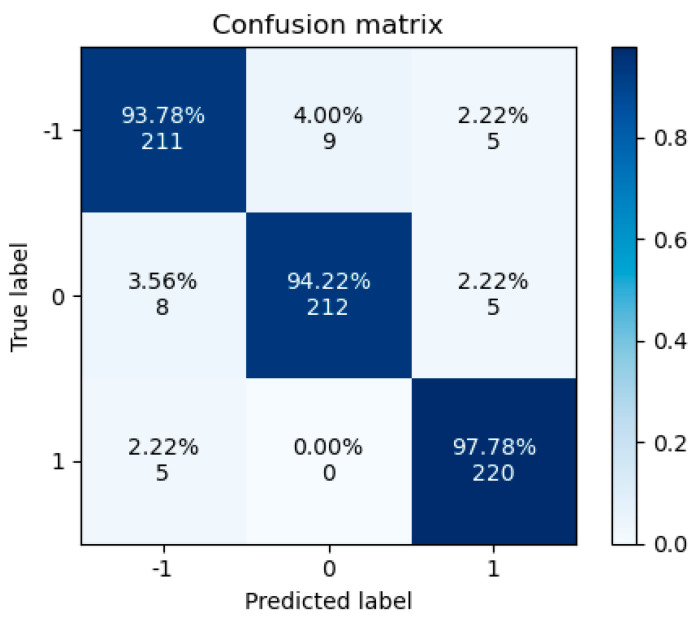
Subject-dependency confusion matrix.

The confusion matrix of the subject-dependent experiment is shown in Figure 6, where the horizontal axis represents the predicted labels and the vertical axis represents the true labels. The labels corresponding to −1, 0, and 1 are negative, neutral, and positive, respectively. The recognition rates for the negative, neutral, and positive emotions were 93.78%, 95.56%, and 96.44%, respectively. Consistent with the results of previous studies, the positive and neutral emotions were easier to recognize than the negative emotions [28]. Note that negative and neutral emotions are more easily confused.

#### 4.2.3. Channel Frequency Band Selection

**Figure 7:**
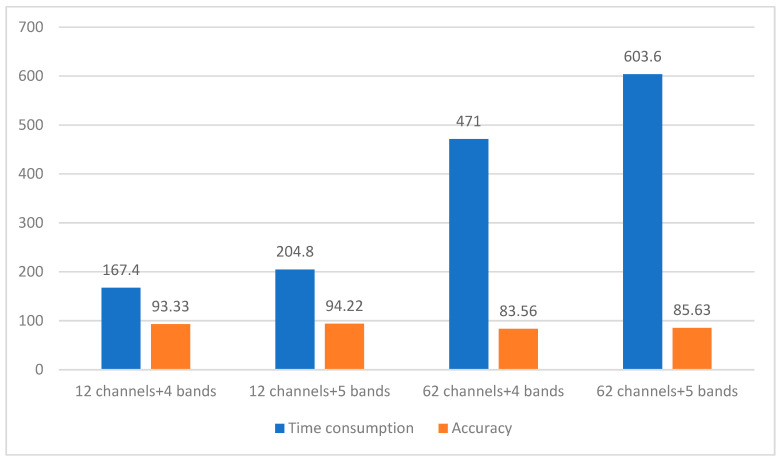
Comparison of time consumption and accuracy.

To compare the effects of the number of channels and frequency bands on the time complexity and accuracy of the proposed method, we conducted an experiment to compare performance when using four and five frequency bands, as well as the combination of 12 and 62 channels. The experimental results are shown in Figure 7, where the blue bar chart represents the time (in ms) consumed for each epoch under this combination condition and the orange bar chart represents the accuracy. From the frequency band perspective, under the same number of channels, the time consumption of five frequency band combinations was 1.22–1.28 times that of the four frequency band combinations, and the accuracy was improved by 1%–2%. These results indicate that the combination of five frequency bands retains more information about emotions. From the channel perspective, under the same frequency band, the time consumption of 62 channels was 2.81–2.95 times that of 12 channels; however, the accuracy decreased by approximately 10%. Thus, using all channels does not improve accuracy. The reduced accuracy may be due to the fact that not all channels of information in the EEG signal are related to emotions, and utilizing all channels introduces noisy data.

#### 4.2.4. Top-k Selection

**Figure 8:**
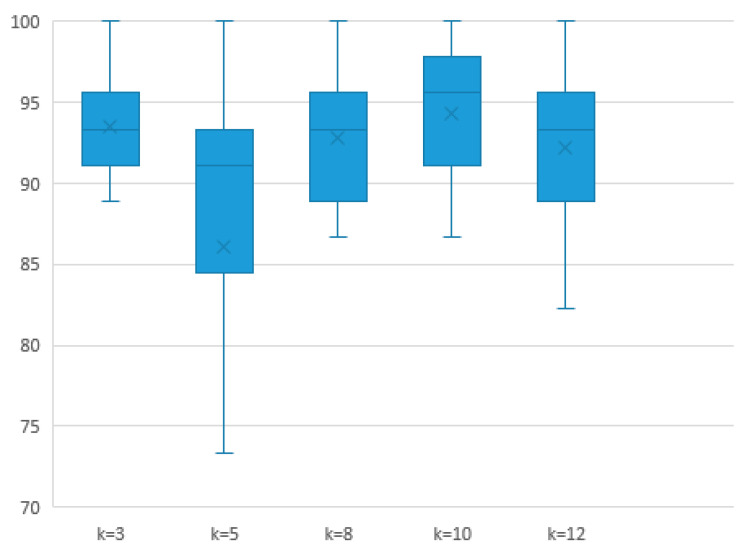
Effect of top-k on the performance of the proposed SOGPCN.

To obtain the sparse adjacency matrix of the graph, we adopted the top-k technique, which involves retaining only the maximum k connection weights of each electrode in the adjacency matrix and setting the remaining weights to 0. In this experiment, we used 12 channels and five frequency bands from the SEED dataset under a single experimental strategy to explore the impact of different top-k values on experimental accuracy. Figure 8 shows the impact of different top-k sparse graphs on the performance of the proposed SOGPCN method. Here, k = 10 indicates that only the maximum 10 connection weights are retained, with all remaining weights set to 0. Similarly, k = 12 indicates that all 12 connections between the electrodes are retained. As can be seen, the model with k = 10 connections performed the best, which further validates the effectiveness of the sparse adjacency matrix model.

#### 4.2.5. Ablation Experiment

Ablation experiments were also conducted on the SEED dataset to validate the effectiveness of the feature extraction modules in the proposed SOGPCN method, i.e., the spatial domain feature extraction and temporal domain feature extraction modules. These experiments involved 12 channels and 5 frequency bands, and they followed a single experimental strategy. As shown in Table 4, the results indicate that removing the self-organizing graph convolution module reduced the average accuracy by 1.92%. Similarly, removing the pseudo-3D convolution module and the partial dot product attention modules resulted in a decrease in average accuracy by 24.15% and 1.19%, respectively. Note that the standard deviation demonstrated improvement. These findings suggest that both the spatial and temporal features of EEG data (being multichannel sequential data) are vital in terms of enhancing the classification results and model stability. In addition, the introduction of time information in the SOGPCN model improved the performance of the model, which also proves that the contribution of time features in EEG emotion recognition tasks is crucial and should not be ignored.

## 5. Conclusions

This paper has proposed an attention-based spatiotemporal convolutional network for the task of EEG-based emotion recognition. The proposed model employs a self-organizing graph convolution module to learn the relationships between channels of EEG emotional signals dynamically in each frequency band. It also extracts time-domain features using pseudo-3D convolution and partial dot product attention. In a set of experimental evaluations, the proposed model achieved advanced performance on the SEED dataset. Based on the experimental results from different subjects, we found that the proposed model demonstrates a certain degree of robustness and excellent recognition accuracy for various subjects. The experimental results also align with the results reported by previous studies that indicate positive and neutral emotions are easier to identify than negative emotions, while negative and neutral emotions tend to be easily confused. Both experimental strategies utilized in this study highlight the closer relationship between the high-frequency band and emotional activity, and the relationship between the low-frequency band and emotional activity may be less relevant for the target task. The results of an ablation experiment also demonstrate the importance of the spatial and temporal features in EEG data.

In the future, we plan to explore methods to fuse data from different frequency bands and further enhance the performance of the proposed model. In addition, during the experimental process, we encountered significant differences in the data of subjects from different SEED datasets, which resulted in fluctuations in experimental accuracy among different subjects. Thus, reducing the feature distribution between different subjects remains a worthwhile topic for exploration.

## Figures and Tables

**Table 1:** Data input and output forms.

Model	Input	Output
Model1	$X_{in}\in R^{batch\_size\times 12\times 5\times 265}$	$X_{out}\in R^{batch\_size\times 12\times 5\times 64\times 1}$
Model2 + Model3	$X_{in}\in R^{batch\_size\times 12\times 5\times 32\times 1}$	$X_{out}\in R^{batch\_size\times 12\times 5\times 11\times 10}$
Model2 + Model3	$X_{in}\in R^{batch\_size\times 12\times 5\times 11\times 10}$	$X_{out}\in R^{batch\_size\times 6\times 2\times 5\times 10}$
Model4	$X_{in}\in R^{batch\_size\times 6\times 2\times 5\times 10}$	$X_{out}\in R^{batch\_size\times 5}$

**Table 2:** Subject-independent classification accuracy on SEED dataset.

	SEED						
Model	δ	θ	α	β	γ	(θ, α, β, γ)	All Bands
SVM	43.06/8.27	40.07/6.50	43.97/10.89	48.63/10.29	51.59/11.83	/	56.73/16.29
TCA [29]	44.10/8.22	41.26/9.21	42.93/14.33	43.93/10.06	48.43/9.73	/	63.64/14.88
SA [30]	53.23/7.74	50.60/8.31	55.06/10.60	56.72/10.78	64.47/14.96	/	69.00/10.89
BiHDM [31]	/	/	/	/	/	/	85.40/7.53
BiDANN-S [32]	63.01/7.49	63.22/7.52	63.50/9.50	73.59/9.12	73.72/8.67	/	84.14/6.87
DGCNN [21]	49.79/10.94	46.36/12.06	48.29/12.28	56.15/14.01	54.87/17.53	/	79.95/9.02
RGNN [14]	64.88/6.87	60.69/5.79	60.84/7.57	74.96/8.94	77.50/8.10	/	85.30/6.72
SOGNN [13]	70.37/7.68	76.00/6.92	66.22/11.52	72.54/8.97	71.70/8.03	/	86.81/5.79
ATDD-LSTM [33]	/	/	/	/	/	/	90.92/1.05
SOGPCN (Our model)	**91.56/3.56**	**91.11/4.94**	**82.81/7.93**	**89.78/5.84**	**90.52/7.41**	**93.33/4.13**	**94.22/3.42**

**Table 3:** Subject-dependent classification accuracy on SEED dataset.

	SEED						
Model	δ	θ	α	β	γ	(θ, α, β, γ)	All Bands
SVM	60.50/14.14	60.95/10.20	66.64/14.41	80.76/11.56	79.56/11.38	/	83.99/9.92
GSVCCA [34]	63.92/11.16	64.64/10.33	70.10/14.76	76.93/11.00	77.98/10.72	/	82.96/9.95
DBN [23]	64.32/12.45	60.77/10.42	64.01/15.97	78.92/12.48	79.19/14.58	/	86.08/8.34
STRNN [35]	80.90/12.27	83.35/9.15	82.69/12.99	83.41/10.16	69.61/15.65	/	89.50/7.63
DGCNN [21]	74.25/11.42	71.52/5.99	74.43/12.16	83.65/10.17	85.73/10.64	/	90.40/8.49
BiDANN [32]	76.97/10.95	75.56/7.88	81.03/11.74	89.65/9.59	88.64/9.46	/	92.38/7.04
RGNN [14]	76.17/7.91	72.26/7.25	75.33/8.85	84.25/12.54	89.23/8.90	/	94.24/5.95
4D-CRNN [36]	/	/	/	/	/	94.74/2.32	/
SOGPCN (Our model)	**88.15/12.90**	**85.33/13.45**	**83.26/4.72**	**90.07/5.37**	**91.11/2.55**	**95.26/2.80**	**95.26/3.52**

**Table 4:** Results of ablation experiment.

Model	SEED
	Leave-One-Subject
SOGPCN	94.22/3.42
-SOG	92.30/4.79
-P-3DCNN	70.07/26.24
-PDPAtt	93.03/4.65

## Data Availability

The open access dataset SEED is used in our study. Its links is as follows, https://bcmi.sjtu.edu.cn/~seed/seed.html (granted on 7 May 2020; accessed on 25 April 2022).
